# MiRNA作为肺癌分子诊断工具的前景与挑战

**DOI:** 10.3779/j.issn.1009-3419.2012.02.12

**Published:** 2012-02-20

**Authors:** William CS CHO, 燕 丁, 娟 南

**Affiliations:** 1 Department of Clinical Oncology, Queen Elizabeth Hospital, Hong Kong; 2 天津医科大学总医院，天津市肺癌研究所，天津市肺癌转移与肿瘤微环境重点实验室; 3 港特别行政区 伊利沙伯医院 临床肿瘤科

**Keywords:** 早期诊断, 肺癌, 小分子RNA, 分子诊断, 预测性标志物, 预后

**“在过去的十年里，miRNA已成为调节基因表达的关键分子之一。它几乎涉及肺癌癌变的每一个进程，包括肿瘤进展、血管新生、侵袭和转移。”**

肺癌是最主要的恶性肿瘤类型，也是全球男性癌症死亡率最高的疾病。对于女性来说，肺癌为第四位最常见恶性肿瘤，其死亡率更高居癌症第二位^[1]^。肺癌预后不良，主要原因为诊断时多为晚期且易复发。通过可靠的早期诊断和预后标志物的应用，全球部分肺癌负担或可得以减轻。。

在过去的十年里，miRNA已成为调节基因表达的关键分子之一。它几乎涉及肺癌癌变的每一个进程，包括肿瘤进展、血管新生、侵袭和转移^[2]^。miRNA芯片的广泛综合利用或可识别一些可作为潜在肿瘤标志物的miRNAs^[3]^。近几年来，一些研究已经有效地研发出用于肺癌分子诊断的miRNA生物标志物^[4]^。本文将探索miRNAs作为肺癌生物标志物的最新研究，并将讨论该热点研究领域的前景与挑战。

## miRNAs在肺癌风险评估中的作用

在人类基因组中单核苷酸多态性是最常见的遗传变异，它们有助于癌症风险的评估^[5]^。一项meta分析探讨了*hsa*-*miR*-*196a2* rs11614913多态性与癌症风险的关系。研究发现携带TC/CC基因型的个体较携带TT的个体患肺癌风险高。该研究结果提示*hsa*-*miR*-*196a2* rs11614913多态性的变异或使个体对肺癌更为易感^[6]^。

## 肺癌早期发现与诊断

通过特定miRNA表达的检测，发展中的肺癌或可先于诊断前数年被发现。在众多样本类型中，因为血液易于通过轻微创伤性方式收集，故对于肿瘤生物标志物检测来说血液为理想的样本类型。研究发现与对照组相比，在早期非小细胞肺癌（non-small-cell lung cancer, NSCLC）的血清样本中*hsa*-*miR*-*1254*和*hsa*-*miR*-*574*- *5p*的表达明显升高。上述两个miRNAs的异常表达可先于临床诊断前数月被检测到，敏感性为82%，特异性为77%，可作为筛查与早期诊断的有效生物标志物^[7]^。通过基于Taqman探针的定量反转录（reverse transcription, RT）-PCR和风险评分分析，一组由10个miRNAs（*miR*-*20a*、*miR*-*24*、*miR*-*25*、*miR*-*145*、*miR*-*152*、*miR*-*199a*-*5p*、miR-221、*miR*-*222*、*miR*-*223*和*miR*-*320*）组成的血清谱亦可准确分辨出NSCLC临床诊断前33个月收集的血清样本。其在非小细胞肺癌检测的曲线下面积、敏感性和特异性分别为0.97、0.93和0.90^[8]^。

**“通过特定miRNA表达的检测，发展中的肺癌或可先于诊断前数年被发现。在众多样本类型中，对于肿瘤生物标志物检测来说，血液为理想的样本类型。”**

从肺癌患者血清中鉴定的miRNA标志物中，聚类分析和自组图谱已识别出一个包含大部分可预测样本的集群。相关性分析显示miRNA表达谱最明显的变化似乎发生在接近诊断的某个时间^[9]^。通过研究早期NSCLC患者和匹配的对照人群的配对血清标本，一个研究小组发现NSCLC患者血清中*miR*-*146b*、*miR*-*221*、*let*-*7a*、*miR*-*155*、*miR*-*17*-*5p*、*miR*-*27a*和*miR*-*106a*的表达明显降低，而*miR*- *29c*的表达却明显升高^[10]^。另有一项调查研究评估了与良性肿瘤患者相比肺癌患者血清中miRNAs的临床相关性。与良性肿瘤患者相比，肺癌患者中*miR*-*10b*、*miR*-*141*和*miR*-*155*的水平明显升高。而且，他们发现血清中*miR*-*10b*的高表达和淋巴结转移有关^[11]^。

研究发现螺旋计算机断层扫描（computed tomography, CT）是诊断无症状肺癌的一个有效的检测方法。借助一项通过每年一次的低剂量螺旋CT对高危人群进行筛查以发现肺癌的大型研究，建立了一个多因素风险预测34-miRNA模型，无症状高风险人群诊断为Ⅰ期NSCLCs的曲线下面积为0.89。这种血清检测或可用于高危人群早期NSCLC的筛查中^[12]^。

## 肺癌预后和预测的miRNA标志物

为了避免不必要的治疗和增强CT筛查对死亡率的影响，侵袭性疾病生物标志物的识别对于定义高风险病变的临床有效预测性标志物至关重要。检测CT筛查发现肿瘤发生前收集到的血浆样品的miRNA表达谱，由9个不同miRNAs（*miR*-*221*、*miR*-*660*、*miR*-*486*-*5p*、*miR*-*28*-*3p*、*miR*-*197*、*miR*-*106a*、*miR*-*451*、*miR*-*140*-*5p*和*miR*-*16*）组成的10个表达比率正确地识别了伴有侵袭性疾病的肺癌患者。该标志物的敏感性为80%，特异性为100%。这些miRNA生物标志物可能有助于避免低风险惰性疾病的过度治疗以及高风险转移性疾病的延误诊断。

另一方面，预测生存期的分子生物标志物的发现和应用也可改善肺癌患者的治疗。研究发现Ⅰ期NSCLC患者血清中*miR*-*223*表达的降低与预后较差密切相关^[10]^。对Ⅰ-ⅢA期肺癌患者进行全基因组Solexa研究，之后进行三套独立的定量RT-PCR评估，发现血清中*miR*-*486*、*miR*-*30d*的高表达以及*miR*-*1*、*miR*-*499*的低表达与NSCLC的不良生存相关。这四个miRNAs在血清中的表达或可作为NSCLC患者总生存期的无创性预测性标志物^[14]^。

越来越多的证据表明肺肿瘤组织中miRNA表达的改变也与肿瘤进展和患者的生存相关。miRNAs的沉默是肺癌变的常见事件，而癌组织中的DNA甲基化在激活肺癌表观遗传miRNAs沉默中起重要作用。一项详细的DNA甲基化分析发现41%的原发性NSCLC病例伴有*miR*-*34b*甲基化，而且一项多因素分析表明*miR*-*34b*甲基化与淋巴浸润显著相关。因此，该miRNA的DNA甲基化可作为肺癌侵袭性表型的生物标志物^[15]^。就侵袭而言，脑转移（brain metastasis, BM）在NSCLC患者中很常见。使用miRNA微阵列分析和定量RT-PCR分析，研究发现*miR*-*328*和*miR*-*330*-*3p*的联合能够正确区分BM阳性和BM阴性的NSCLC患者，准确率达80%（敏感性为0.75，特异性为0.82）。这两个miRNAs或可用于临床治疗决策以区分BM高风险的NSCLC患者^[16]^。

**“肺癌组织分析也表明miRNAs可作为肺癌分类、预后分层和药物反应预测的有效分子标志物。”**

*miR*-*21*在多种癌症类型中是致癌性miRNA。通过定量RT-PCR检测NSCLC患者组织中的miRNAs表达，研究发现*miR*-*21*的高表达与Ⅰ期肺腺癌患者的疾病进展呈正相关，与无复发生存呈负相关。该miRNA可作为肺腺癌的早期预后生物标志物^[17]^。另一方面，研究认为*miR*-*126*与肿瘤血管新生和VEGF-A有关。通过高通量组织芯片、原位杂交和免疫组织化学方法检测Ⅰ-ⅢA期NSCLC患者切除的肿瘤组织样本，发现*miR*-*126*和VEGF-A的共表达对5年生存率具有显著的预后影响。研究认为在NSCLC中该miRNA是一个强且独立的负性预后因素^[18]^。

NSCLC患者的表皮生长因子受体（epidermal growth factor receptor, EGFR）突变与EGFR酪氨酸激酶抑制剂（tyrosine kinase inhibitor, TKI）的敏感性密切相关。我们的初步研究发现，*miR*-*145*的上调是控制肺腺癌细胞增殖的一个重要的基因调控机制，且与EGFR的下调密切相关。*miR*-*145*作为EGFR-TKI作用的预测性生物标志物值得进一步研究，并有可能促进肺腺癌患者的个体化EGFR-TKI治疗^[19]^。

## 结论与挑战

miRNA标志物发现的日益增加为癌症分子诊断提供了令人鼓舞的机会^[20]^。肺癌患者血液中miRNAs标志物的鉴定正在快速开展，这可能对无创性早期诊断和随访观察有一定价值。肺癌组织分析也表明miRNAs可作为肺癌分类、预后分层和药物反应预测的有效分子标志物。

尽管近年来肺癌中miRNA标志物的鉴定取得了可喜的成果，但仍无一标志物得以进入临床阶段。大多数用于早期检测的标志物的敏感性和特异性水平似乎还不尽人意，miRNA表达分析的检测和定量技术也需要改善。用于区分早期恶性和非恶性肿瘤的miRNA标志物有待大力发展。没有临床易用并经济有效的miRNA检测技术，miRNAs就不能直接影响肺癌的治疗。

对于肺癌的预后和预测性标志物，尽管在众多研究中病例与对照组之间存在明显的统计学差异，这些令人振奋的结果应用到临床实践仍需要合理的验证。充分认识并定义常规程序、标准以及质控从而将宝贵的实验结果转化为肺癌患者的临床相关程序非常必要。须特别关注的是用以解决标志物的临床问题的周密的系列设计和大型多中心临床试验的执行。如果设计严谨且严格遵守良好的临床实践，这些试验有可能会使新兴的生物标志物用于临床实践。到底miRNA标志物能否转化为肺癌的临床实践，结论为时过早，但大量的有关miRNAs用于肺癌筛查、预后和监测抗癌治疗疗效的潜在应用的信息无疑已经涌现。

## Financial & competing interests disclosure

*The author has no relevant affiliations or financial involvement with any organization or entity with a financial interest in or financial conflict with the subject matter or materials discussed in the manuscript*. *This includes employment*, *consultancies*, *honoraria*, *stock ownership or options*, *expert testimony*, *grants or patents received or pending*, *or royalties*.

*No writing assistance was utilized in the production of this manuscript*.
